# Consistency and quality in written accreditation protocols for pediatrician training programs: a mixed-methods analysis of a global sample, and directions for improvement

**DOI:** 10.1186/s12960-023-00852-2

**Published:** 2023-08-17

**Authors:** Alexandra L. Coria, Areej Hassan, Jui-Yen Huang, Katia C. Genadry, Rashmi K. Kumar, Ayten Sergios, Roseda E. Marshall, Christiana M. Russ

**Affiliations:** 1 SUNY Downstate College of Medicine, 450 Clarkson Ave Suite J, Brooklyn, NY 11203 USA; 2 Department of Population Health, Maimonides Medical Center and Division of Hospital Medicine, Maimonides Children’s Hospital, 4802 10th Ave, Brooklyn, NY 11219 USA; 3 Harvard Medical School, 25 Shattuck St, Boston, MA 02115 USA; 4 Division of Adolescent/Young Adult Medicine, Boston Children’s Hospital, 300 Longwood Avenue, Boston, MA 02115 USA; 5 Division of Adolescent Medicine, Department of Pediatrics, Kaohsiung Medical University Hospital, Kaohsiung Medical University, No. 100, Tzyou 1st Rd., Sanmin Dist., Kaohsiung City, 80756 Taiwan, ROC; 6 Division of Emergency Medicine, Boston Children’s Hospital, 300 Longwood Avenue, Boston, MA 02115 USA; 7 University of Nairobi, P.O Box 30197, GPO, Nairobi, Kenya; 8 Kenyatta National Hospital, P.O Box 20723-00202, Nairobi, Kenya; 9 AM Dogliotti College of Medicine, Capitol Hill, P.O Box 10-9020, 1000 Monrovia 10, West Africa Liberia; 10 Intermediate Care Program, Boston Children’s Hospital, 300 Longwood Avenue, Boston, MA 02115 USA

**Keywords:** Accreditation, Post-graduate medical education, Medical education research, International medical education, Global pediatrics

## Abstract

**Background:**

The World Federation for Medical Education (WFME) defines accreditation as 'certification of the suitability of medical education programs, and of…competence…in the delivery of medical education.' Accreditation bodies function at national, regional and global levels. In 2015, WFME published quality standards for accreditation of postgraduate medical education (PGME). We compared accreditation of pediatric PGME programs to these standards to understand variability in accreditation and areas for improvement.

**Methods:**

We examined 19 accreditation protocols representing all country income levels and world regions. For each, two raters assessed 36 WFME-defined accreditation sub-areas as present, partially present, or absent. When rating “partially present” or “absent”, raters noted the rationale for the rating. Using an inductive approach, authors qualitatively analyzed notes, generating themes in reasons for divergence from the benchmark.

**Results:**

A median of 56% (IQR 43–77%) of WFME sub-areas were present in individual protocols; 22% (IQR 15–39%) were partially present; and 8.3% (IQR 5.5–21%) were absent. Inter-rater agreement was 74% (SD 11%). Sub-areas least addressed included number of trainees, educational expertise, and performance of qualified doctors. Qualitative themes of divergence included (1) variation in protocols related to heterogeneity in program structure; (2) limited engagement with stakeholders, especially regarding educational outcomes and community/health system needs; (3) a trainee-centered approach, including equity considerations, was not universal; and (4) less emphasis on quality of education, particularly faculty development in teaching.

**Conclusions:**

Heterogeneity in accreditation can be appropriate, considering cultural or regulatory context. However, we identified broadly applicable areas for improvement: ensuring equitable access to training, taking a trainee-centered approach, emphasizing quality of teaching, and ensuring diverse stakeholder feedback.

## Background

In 2010, the Lancet Commission on Education of Health Professionals for the twenty-first century called for strengthening medical education resources globally [1]. Since then, international organizations like the World Health Organization (WHO) and the World Federation for Medical Education (WFME) have become increasingly invested in improving and standardizing medical education as a means to improve equitable access to high-quality health care globally. One means of improving education is accreditation, defined as “the certification of the suitability of medical education programs, and of the competence of medical schools in the delivery of medical education.” [2]. In a 2016 study, 91 (84%) of 108 countries with pediatric training programs reported some available accreditation agency [3]; of those, 8 relied on regional agencies [West African College of Physicians, Arab Board or Conseil Africain et Malgache pour l’Enseignement Superieur (CAMES)], 55 were national medical organizations and 21 were ministries of health or education. The remaining 7 countries had no nationally centralized accreditation process [4]. Since 2010 the Accreditation Council for Graduate Medical Education-International (ACGME-I) has extended accreditation to individual institutions or through government ministries and university systems. Institutions, learners and individual physicians generally view accreditation as a useful and positive exercise [5, 6].

As accreditation of medical training programs becomes more common and valued, best practices are emerging. One major best practice guide for postgraduate medical education (PGME) was published by the WFME in 2015 and revised in 2023 via a consensus-based approach among global medical education experts [7, 8]. Some work has been done looking at variability in accreditation of surgical training programs [9, 10], and a 2021 scoping review in LMICs examined barriers to implementation of the separate undergraduate WFME accreditation standards. Challenges at the undergraduate level included leadership and governance, engagement of students as stakeholders, and curricular development [11]. However, there has been very little comparison of accreditation protocols to the WFME PGME recommendations, and none specifically in pediatrics. This study seeks to understand how accreditation of pediatric PGME programs varies globally in comparison to the WFME standard, and to identify common targets for international efforts to improve and standardize pediatric PGME through accreditation.

## Methods

We sourced written accreditation protocols from 19 national, regional and global PGME accreditation bodies. We initially employed a purposive sampling strategy: we identified countries in each WHO Region that had indicated in the 2016 study they had a national accreditation agency, and had two or more pediatric PGME programs. We prioritized countries that train a large number of pediatricians. We also identified major regional and international accreditation bodies. In WHO Regions where the medical systems were likely to be modeled on those of former colonizers, we deliberately included countries to reflect multiple colonial histories. We limited our search to protocols in languages spoken by at least one member of the author team including English, Spanish, French, Chinese and Arabic.

Although accreditation protocols are often not publicly accessible [12], we searched accrediting organizations’ websites for documents describing their protocol. If that failed, we attempted to obtain the documents by contacting the organization and emailing our professional networks. Sampling was stopped when every geographic region and World Bank income level was represented (Table 1).

**Table 1:** Diversity of sampled accreditation protocols

Accreditation catchment area	World Bank income level	WHO region	Continent	Language of accreditation protocol	Pediatric-specific?
All countries except United States	All	All	All	English	Yes
Arab countries	All	Eastern Mediterranean/ African	Africa/Asia	Arabic and English	Yes
Argentina	Upper-middle	Americas	South America	Spanish	No
Australia	High	Western Pacific	Australia	English	No
Bhutan	Lower-middle	South-East Asian	Asia	English	No
Canada	High	Americas	North America	English	Yes
China	Upper-middle	Western Pacific	Asia	Chinese	Yes
Colombia	Upper-middle	Americas	South America	Spanish	No
Ethiopia	Low	African	Africa	English	Yes
Hong Kong	High	Western Pacific	Asia	English	Yes
India	Lower-middle	South-East Asian	Asia	English	Yes
Ireland	High	European	Europe	English	No
Kenya	Lower-middle	African	Africa	English	No
Saudi Arabia	High	Eastern Mediterranean	Asia	Arabic and English	Yes
Switzerland	High	European	Europe	French	Yes
Taiwan	High	Western Pacific	Asia	Chinese	Yes
United Kingdom	High	European	Europe	English	No
United States	High	Americas	North America	English	Yes
West Africa	Upper-middle, lower-middle, low	African	Africa	English	Yes

### Quantitative analysis

**Fig. 1:**
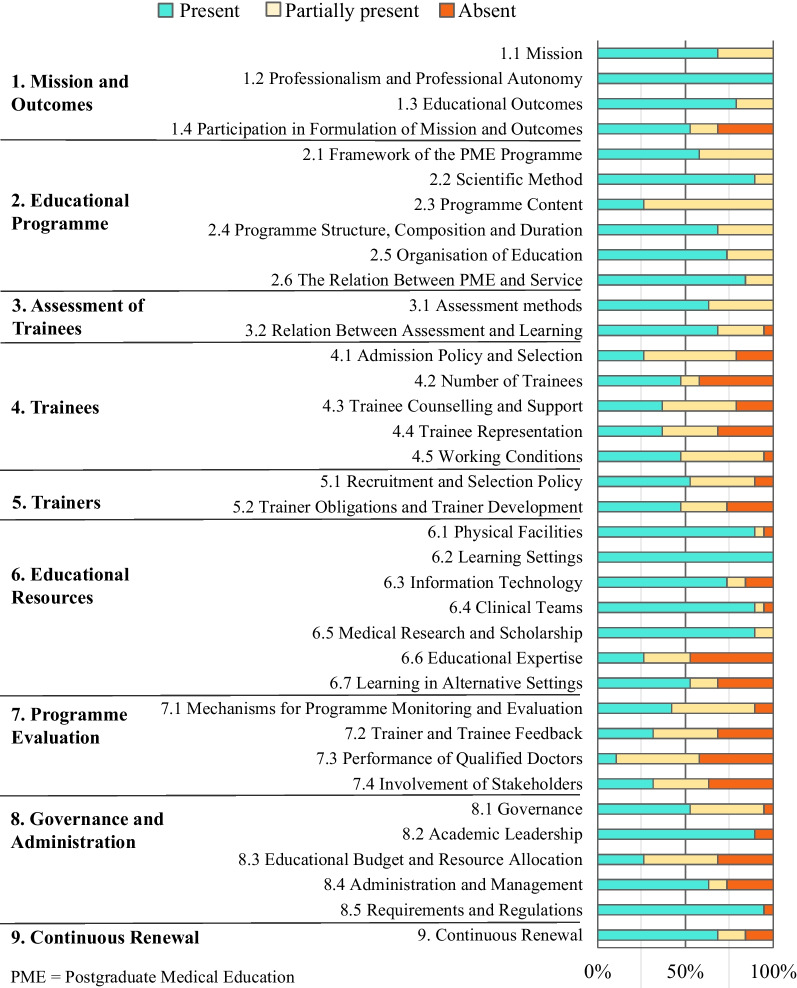
Percent of accreditation protocols (*n* = 19) addressing 36 sub-areas of a global benchmark accreditation standard

We assessed each sampled protocol for presence of the elements described in the WFME benchmark document, which is divided into 9 Areas and 36 sub-areas (Fig. 1). Within each sub-area, the WFME describes quality standards in PGME accreditation practices. The WMFE specifies two levels of standard for each sub-area: “basic” and “quality development”. We looked only for language addressing “basic” standards, which reflect minimum quality recommendations and contain between one and ten described practices.

Two raters independently assessed each sub-area as present, partially present, or absent in each sampled protocol by looking for language that indicated that WFME-recommended standards were part of the accreditation process. They met to reconcile differences in ratings by jointly reviewing the protocols. A third rater was consulted if agreement could not be reached. Protocols available in an official English version were analyzed in English. For non-English protocols, two fluent speakers of the document’s language conducted the quantitative comparison analysis. When a third rater was needed, a fluent speaker of both languages translated the relevant parts of the document into English.

We compiled data and conducted quantitative analysis in Google Sheets (Google, Mountain View, CA, USA) and Microsoft Excel (Microsoft, Seattle, WA, USA). Means were compared using Student’s *t*-tests. Inter-rater agreement was calculated as a percentage of initial ratings that were the same.

### Qualitative analysis

If they categorized a WFME sub-area as “partially present” or “absent”, raters wrote comments in English describing the specific inconsistency with WFME standards. Using an inductive approach, authors AC and CR qualitatively coded these comments to identify themes in the reasons for divergence from the WFME benchmark.

They used open coding, independently creating and assigning codes to each comment. After coding the first 11 protocols, they met to verbally reconcile codes. They continued to independently code and reconcile codes until comments on all sampled protocols were coded. Subsequently all data were re-coded according to agreed-upon defined codes, and all codes were reconciled.

Coders then independently drafted inductive themes and met to reconcile them. The full author team, who work in a diversity of cultures and health systems, then reviewed the final coding and preliminary themes and came to consensus on final themes.

## Results

Nineteen accreditation protocols were examined. Twelve documents were written in English; two in Spanish; two in Chinese, two in Arabic, and one in French (Table 1). Format of the protocols ranged from a single 12-page document to a set of related documents exceeding 50 pages altogether.

### Quantitative results

Figure 1 summarizes results of the assessment for presence of the 36 sub-areas of the WFME benchmark. Raters agreed 74% of the time (SD 11%), with all discrepancies verbally resolved.

**Fig. 2:**
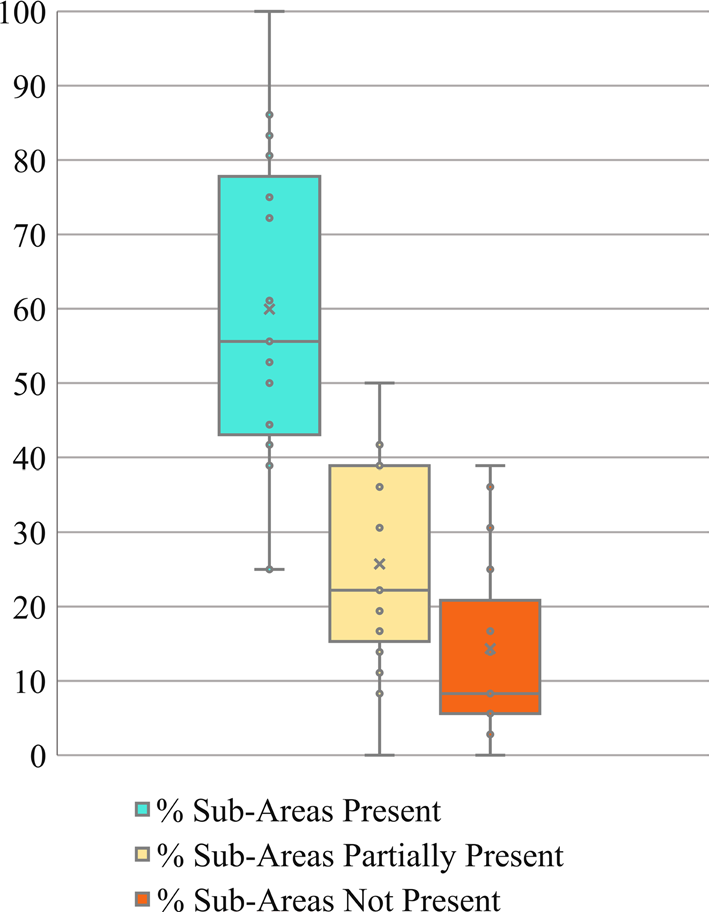
Percent of WFME-endorsed accreditation standards present in each of 19 accreditation protocols. The WFME organizes suggested standards into 36 sub-areas, rated as “present”, “partially present” or “not present” by the study team

For individual accreditation bodies’ protocols, a median of 56% (IQR 43–77%) of WFME sub-areas were rated present; 22% (IQR 15–39%) were rated partially present; 8.3% (IQR 5.5–21%) were rated absent. Figure 2 shows the range of results by protocol, for example one protocol had only 25% of sub-areas rated present, while another included 100% of sub-areas.

Only two sub-areas were fully addressed in all 19 protocols: 1.2: professionalism and professional autonomy and 6.2: learning settings. Sub-areas least likely to be present or partially present were 4.2 number of trainees, 6.6 educational expertise, and 7.3 performance of qualified doctors. Sub-areas least often coded as fully present to a level of quality matching that outlined in the WFME benchmark document were 2.3 program content, 4.1 trainee admission policy and selection, 6.6 educational expertise; 7.3 performance of qualified doctors; and 8.3 educational budget and resource allocation (Fig. 1).

There was no statistical difference in presence of sub-areas between protocols that were pediatric-specific (63% of the sample) and specialty-nonspecific protocols (27%).

Due to small sample size and the fact that some accreditation bodies function regionally and globally, we could not compare agreement with the WFME benchmarks across WHO regions or country income levels.

### Qualitative results

We reached saturation of qualitative codes after coding comments on 15 protocols. Several themes emerged that describe patterns in the disagreement between our sampled accreditation protocols and the standard set by the WFME. These are:

1. Variation in accreditation protocols is related to heterogeneity in structure, composition and duration of the PGME programs they accredit.
  Coded comments reflected the following patterns:
  WFME-recommended content areas were frequently absent; specific examples include medical jurisprudence, forensics, complementary medicine and doctor self-care.
  There was heterogeneity in whether accreditation protocols explicitly required that PGME programs remunerate trainees, specify expected duration of training or define the number of accepted trainees.
  Two protocols did not specify which parts of the training program would be compulsory versus optional.
2. Engagement with WFME-specified stakeholders is limited, with lack of feedback from stakeholders about long-term educational outcomes and community and health system needs. WFME defines “principal stakeholders” in PGME programs as “trainees, programme directors, medical scientific societies, hospital administrations, governmental authorities, other health care authorities and professional associations or organisations as well as representatives of supervisors, trainers and teachers. Some principal stakeholders may be programme providers as well [7].” References to stakeholder involvement are present in many sub-areas, including within areas 1. mission and outcomes, 2. educational program, and 7. program evaluation.
  Coded comments reflected the following patterns:
  Sixteen accreditation protocols mentioned none or only a subset of the WFME-defined “stakeholders”, contributing heavily to “partially present” and “not present” ratings.
  Only one of the 19 standards required feedback from trainees and/or trainers in design, improvement and monitoring of the program.
  Fourteen protocols did not require a mechanism for feedback on professional performance of a program’s graduates, other than a final exit exam.
  Six protocols did not require integration of the specific needs of the local community into the mission, outcomes or design of a training program.
3. A trainee-centered approach is not universal, including equity considerations and a supportive training environment.
  Coded comments reflected the following patterns:
  Most (15/19) examined protocols were missing one or more WFME-recommended standards meant to protect the health and well-being of trainees, such as policies on managing interruptions in training, providing supportive counseling services, or ensuring an appropriate service-learning balance.
  Three protocols did not require a mechanism for trainees to appeal assessments.
  For sub-areas that set standards around equitable access to training (such as 2.1 framework of the PME program and 4.1 admission policy and selection), 12 accreditation bodies did not require policies to ensure equitable and transparent selection of trainees. Six comments specifically noted that there was no mention of protections for individuals with disabilities.
4. There is less emphasis on ensuring quality of education, particularly faculty development in teaching.
  Coded comments reflected the following patterns:
  Nine protocols received “not present” ratings on sub-area *6.6 educational expertise*, which sets out three areas (program planning, implementation and evaluation) in which the WFME recommends that educational experts be consulted. Five more protocols required teacher training but no expertise in educational design or assessment.
  In eight accreditation protocols, comments noted no requirement for trainers to have protected time for teaching or mentorship.
  Nine protocols did not lay out expectations around faculty assessment or training as teachers.

## Discussion

The WFME states in the introduction to the 2015 standards that, “Each institution or regulator should…develop a version of [this benchmark standard] that is appropriate to the local context…Not all standards may have application in every setting [7].” Our findings begin to describe the high degree of variability that currently exists in the standards to which pediatric PGME programs are held, depending on which body accredits them. We hope this study will help explore what constitutes appropriate contextual variation in accreditation, while still ensuring a high global quality of pediatric PGME.

It is also important to note that in 2023 the WFME published a highly revised set of “principles-based” standards that, rather than suggesting quality measures that could serve as checkboxes, suggests questions for the accreditation body to ask when designing its protocol [8]. Below, we discuss our findings in the context of this revised guidance.

### What constitutes contextually appropriate variability in accreditation?

Recent work published by Harper, et al. [13], quantified the wide flexibility in the role of the pediatrician from country to country, a specific example of contextual variability that may affect accreditation requirements around program content. In our analysis we noted WFME-specific content areas of “interface with complementary medicine” or “medical jurisprudence” were frequently the missing elements that led to a “partially present” rating in sub-area 2.3: program content. These are content areas that may be highly influenced by cultural, legal and political context; leaving them out of a curriculum may not impair physician competence in their practice context.

Heterogeneity in the structure, composition or duration of PGME programs also contributed to the reasons that accreditation protocols were inconsistent with the WFME standards. In some cases, the accrediting body omitted guidance on duration of pediatric PGME programs, how many trainees could be admitted each year, which parts of a program were compulsory versus optional, or the exact structure of the program. These might be areas where the accreditation body could strengthen its oversight, or they might be contextually appropriate; for example, in the Swiss system we examined, PGME programs are highly decentralized. Trainees find a series of paid placements that meet certain requirements; because of this structure, requirements around number of trainees or duration of training may not be appropriate or feasible. Indeed, another decentralized system in Germany recently used the 2015 WFME standards to write its own context-appropriate accreditation guiding document, purposefully leaving out sub-areas over which they did not have organizational control [14].

Notably, our methodology did not assess which sampled protocols were *more* rigorous or specific than the WFME benchmark, although we did note this to be the case a number of times. For example, in some lower-income countries, accreditation protocols included detailed lists of required hospital equipment, access to which may have been assumed in higher-income health systems.

### Greater latitude in the 2023 WFME standards

In acknowledgement of the difficulty in setting out one guiding document for the world, the 2015 WFME standards are agnostic to many features of an accreditation body, for example whether it is government-affiliated, the structure of the health or training system it serves, and many specific practices, such as suggesting that the accreditation body ensure that trainees see “a relevant number of patients”, without a minimum number [7]. With the 2023 standard, the WFME goes further in this agnosticism, providing question prompts for the accreditation body to consider. For example, it asks accreditors to consider, “How does the responsible body ensure consistency of curriculum delivery and practical experience in workplace settings?” It would now be impossible to conduct a quantitative, checkbox-style analysis replicating the current one because, as the WFME says in its introduction to the 2023 document, “[The new standards] require thought and discussion, so they deter a shallow or instrumental compliance response. It is hoped that they might trigger a deep analysis of the postgraduate medical education process [8].”

While there is value in this new method that benefits application to heterogeneous contexts, a potential unintended consequence may be perpetuation of certain biases that marginalize less powerful groups in the design and evaluation of medical training. Our analysis found that there was very uneven attention paid to issues that would be important to trainees, including: guaranteeing applicants an equitable selection process regardless of gender, ethnicity or disability; soliciting trainee feedback on the program itself; ensuring flexibility in case of a necessary training interruption (such as maternity, medical or bereavement leave); emphasizing physician self-care and a manageable balance of service and learning; and building in career mentorship. These elements of a training program prioritize the wellness and learning of trainees, and should be built into any robust accreditation process, as research increasingly links the wellness of practicing medical trainees to improved outcomes and patient safety [15, 16].

For example, the 2015 standard enumerates in sub-area 4.1: trainees: admission policy and selection that “The programme provider(s) must…formulate and implement a policy on: the criteria and the process for selection of trainees, [including] admission of trainees with disabilities requiring special facilities…[7]” The 2023 document approaches this recommendation differently, making the recommendation that admissions bodies have publicly available admissions policies, including detailing the “numbers and locations of…posts, equality, equity, inclusivity, and diversity issues.” The associated self-assessment questions are: “How is the selection and progression policy designed to be fair and equitable within the local context? How are issues of equality, equity, inclusivity, and diversity addressed [8]?” While we agree that freedom to consider cultural norms and context is very important in this guiding document, there are data from the United States (though a notable dearth from anywhere else) that suggest that a deliberate approach to diversity initiatives in PGME admissions is important in ensuring equitable access to training [17]. Recruiting a diverse workforce may in turn have benefits for patient safety, experience and outcomes [18–20]. Diluting recommendations about inclusivity and diversity may hinder progress toward equity in admissions to PGME programs.

Accreditation bodies need latitude to consider local context in their processes, but our analysis indicates that they may also benefit from specific tools, like checklists, with which to grapple with biases inherent in their medical training systems and to address evidence-based best practices like diversity and trainee wellness initiatives that may be in their infancy in the local context. This need is also supported by previous work looking at barriers to implementation of WFME standards in undergraduate medical education contexts [11].

### The role of stakeholders in PGME accreditation

In 2015, the WFME specified a group of “principal stakeholders” whom they recommend be engaged in every level of program evaluation from mission development to long-term evaluation of trainees. Although literature supports that PGME program directors themselves identify close coordination with stakeholders a critical part of their job [21]**,** only three analyzed protocols met the standard of requiring engagement of all specified principal stakeholders. The 2023 standard has largely removed this list, referencing consultation with stakeholders more generally. The WFME’s 2015 list may be useful, however, as a menu of options, each of which should be carefully evaluated as a potentially important stakeholder in a particular context. Where the 2015 standard did not include in its list of principal stakeholders the communities and patients served by the physicians in the training programs (despite emphasizing in other places the importance of responsiveness to the community), the 2023 document specifically does reference the community as an important PGME stakeholder. This change may reflect a response to patient and family centered care movements which call increasingly for family and community involvement in medical education [22–24].

This disconnect between programs and stakeholders is perhaps best exemplified by how few accreditation bodies had any requirement to monitor performance of fully qualified doctors or seek feedback from employers. This lack of long-term outcomes monitoring has recently been brought to the fore by Phillips, et al., who highlighted challenges and some potentially promising methods for measuring graduate medical education outcomes on individual, institutional and societal levels. They also note that this monitoring is necessary to honor medicine’s social contract, a conclusion with which we agree [25]. While this article is specific to the United States, the global community shares the challenge of collecting and sharing meaningful data to inform not just training processes but also to direct and justify financial investments in graduate medical education. Ultimately, ensuring that the pediatric workforce can address child health needs in their communities does require engagement with a broad set of stakeholders. Some of these stakeholders may need the weight of accreditation requirements to ensure their less powerful voices are included.

### Need for a focus on quality of medical education

In our qualitative analysis, we often noted lack of required faculty development in teaching, dedicated faculty time for education (versus service or research), or the engagement of experts in education for designing or evaluating the program. In a 2016 study of clinical educators in programs accredited by ACGME-I, they reported little or no perceived value for teaching or educational activities in their institutions; further, only 44% felt competent in curriculum development, and 32% in educational research [26]. Another study of ACGME-I accredited institutions found that turnover is high among program directors in newly accredited institutions globally [27], lending even more urgency to the need for high-quality professional training resources for medical educators in recently accredited institutions.

Improving the quality of teaching and educational practices by emphasizing them through the accreditation process is, we find, an area for broad improvement in our sampled protocols.

### Study strengths and limitations

This study brought together a diverse author group in terms of experience, background and country of practice. Our mixed-methods approach allowed us to examine patterns in our quantitative data more deeply and begin to address the question of where the WFME and other international bodies might specifically consider the appropriateness of making certain recommendations (e.g., which stakeholders to consult), or might consider providing capacity enhancement support (as with designing diversity initiatives or faculty development plans).

We acknowledge, however, that our small sample limited the quantitative part of our analysis, specifically in our ability to compare results across economic, cultural, linguistic and other categories, including ones important to accreditation and medical training, such as affiliation of the accrediting body, structure of the health system, legal requirements for accreditation, and data sources used in accreditation evaluations. We were limited by difficulty sourcing accreditation documents [a known problem in the medical education literature [12]], as well as by the resource-intensive nature of the analysis. We also acknowledge that our sample is weighted toward high-income settings, and the quantitative conclusions may reflect that bias. However, as we did reach saturation of codes and themes in our qualitative analysis, we believe that it can robustly inform future efforts by the WFME and others to improve accreditation processes globally, and guide future research efforts.

A second limitation of our study is that because the only information available to us was the written accreditation protocols, there may be assumptions or cultural norms that we cannot understand from written documents alone, and it would be impractical to consult context experts on the interpretation of all studied accreditation protocols.

Finally, there is a an extra—and possibly under-appreciated—layer of complexity that emerged during discussions among our international author group, that both highlights the need for robust intercultural dialogue on what constitutes high-quality PGME, and may present a limitation to our study. During our analysis, we identified that we did not have a shared understanding of certain terms—for instance, whether “community” stakeholders referred to the public community served by a health system or the community of healthcare providers within the system. Even if accreditation bodies write protocols that appear to be similar, the ultimate interpretation of those protocols is influenced by cultural and linguistic context as well as other factors.

## Conclusions

This study furthers understanding of how accreditation of pediatric PGME programs varies globally, and identifies targets for international efforts to improve and standardize pediatric PGME through accreditation. While all practices enumerated in the 2015 WFME standards may not be appropriate for all contexts, there may be merit in more specificity when it comes to ensuring equitable access to training, policies protective of trainee well-being and autonomy, stakeholder engagement, and valuing and incorporating educational expertise, which were areas where many of the protocols we examined were lacking. We believe that where there is high-quality evidence for best practices in these areas, there is a role for specific, evidence-based guidance from the WFME. Further, there is a role for the WFME or other international bodies to facilitate opt-in capacity building initiatives to accreditation bodies and PGME programs to provide expertise lacking in these areas. More investigation is also needed to establish these best practices in more diverse settings, with resource sharing and advocacy at an international level.

Moreover, there is a dearth of evidence on how accreditation processes and specific elements of accreditation impact educational outcomes, and ultimately the health care provided by graduates to their communities. As accreditation, especially first-time accreditation, is known to be challenging but thought to be worthwhile [5, 6, 21], how accreditation variability translates into practice variability is an enormous and important area for future research.

Finally, the global community must continue to engage in a robust dialogue on what constitutes appropriate influence of culture and context on variability in accreditation practices. The revised 2023 WFME standards reflect that that dialogue has been ongoing [8]. We hope that our analysis will inform the next iteration of that document, as well as the way that accreditation is used as a tool to promote high-quality health care for all the world’s children.

## Data Availability

The datasets used and/or analyzed during the current study are available from the corresponding author on reasonable request.

## Abbreviations

ACGME-I: Accreditation Council for Graduate Medical Education-International
LMICs: Lower- and middle-income countries
PGME: Post-graduate medical education
WFME: World Federation for Medical Education
